# Non-clinical interventions to reduce unnecessary caesarean sections: WHO recommendations

**DOI:** 10.2471/BLT.19.236729

**Published:** 2019-11-29

**Authors:** Newton Opiyo, Carol Kingdon, Olufemi T Oladapo, João Paulo Souza, Joshua P Vogel, Mercedes Bonet, Maurice Bucagu, Anayda Portela, Frances McConville, Soo Downe, Ahmet Metin Gülmezoglu, Ana Pilar Betrán

**Affiliations:** a UNDP/UNFPA/UNICEF/WHO/World Bank Special Programme of Research, Development and Research Training in Human Reproduction (HRP), Department of Reproductive Health and Research, World Health Organization, Avenue Appia 20, 1211 Geneva 27, Switzerland.; b School of Community Health and Midwifery, University of Central Lancashire, Preston, England.; c Department of Maternal, Newborn, Child and Adolescent Health and Ageing, World Health Organization, Geneva, Switzerland.

Caesarean sections can be a lifesaving procedure for mother and baby, but rates beyond 10% of live births are not associated with reductions in maternal and newborn mortality.^1^ Caesarean section rates at national level vary between around 2% in Chad, Burkina Faso, Ethiopia or Madagascar and above 50% in Brazil, Dominican Republic or Egypt.^2^ The trend towards overuse of caesarean sections is a major concern globally, given the risks to the mother and her child associated with unnecessary caesarean birth. These risks include avoidable maternal complications such as infections, haemorrhage, complications related to use of anaesthesia or blood transfusion, and infant morbidity, for example, respiratory problems, asthma and obesity in children. Caesarean sections can also lead to added complications for the mother in subsequent pregnancies, including uterine rupture, placental implantation problems and need for hysterectomy.^3^ High rates of caesarean sections are also associated with substantial health-care costs, which can pose a considerable burden on health systems.

Multiple factors are driving increases in caesarean section rates. Clinical reasons for growing rates include increases in the incidence of maternal obesity, multiple pregnancies and a higher maternal age at birth. These factors alone are unlikely to explain the extent of the rise in caesarean section rates or the substantial variations among health-care providers, hospitals and regions. Studies have shown associations between caesarean section rates and non-clinical factors such as differences in health provider practices, fear of malpractice litigation and organizational, economic, social and cultural factors.^4^ A growing proportion of caesarean sections globally are not medically indicated and could have been avoided.

To address the rising rates worldwide and prevent the harm to women and newborns resulting from overuse of this procedure, in 2018 the World Health Organization (WHO) published new recommendations on non-clinical interventions to reduce unnecessary caesarean sections.^5^ In this guideline, non-clinical interventions are defined as those interventions that are applied outside of the routine clinical interactions between a provider and pregnant woman. The interventions may target women (for instance, birth preparation classes), health-care providers (clinical practice guidelines) or health organizations (different payment systems for caesarean sections).

The recommendations (Box 1), are grouped according to the target of interventions and address major determinants of caesarean section rates. The recommendations are intended to inform the development of national and subnational policies and protocols to reduce unnecessary caesarean births in high-, middle- and low-income countries. The new recommendations should be integrated and implemented with other related WHO guidelines, such as WHO recommendations on antenatal and intrapartum care for a positive childbirth experience.

Box 1 **Summary list of WHO recommendations on non-clinical interventions to reduce unnecessary caesarean sections**

**Interventions targeted at women**

Recommendation 1. Health education for women is an essential component of antenatal care. The following educational interventions and support programmes are recommended to reduce caesarean births only with targeted monitoring and evaluation. *(Context-specific recommendation, low-certainty evidence)*

- Childbirth training workshops: Content includes sessions about childbirth fear and pain, pharmacological pain-relief techniques and their effects, non-pharmacological pain-relief methods, advantages and disadvantages of caesarean sections and vaginal delivery, indications and contraindications of caesarean sections, among others.
- Nurse-led applied relaxation training programme: Content includes group discussion of anxiety and stress-related issues in pregnancy and purpose of applied relaxation, deep breathing techniques, among other relaxation techniques.
- Psychosocial couple-based prevention programme: Content includes emotional self-management, conflict management, problem solving, communication and mutual support strategies that foster positive joint parenting of an infant. Couple in this recommendation includes couples, people in a primary relationship or other close people.
- Psychoeducation for women with fear of pain: Content includes information about fear and anxiety, fear of childbirth, normalization of individual reactions, stages of labour, hospital routines, birth process, and pain relief led by a therapist and midwife, among other topics.

When considering the educational interventions and support programmes, no specific format (pamphlet, videos, role play education) is recommended as more effective. *(Low- to moderate-certainty evidence)*

**Interventions targeted at health-care professionals**

Recommendation 2.1. Implementation of evidence-based clinical practice guidelines combined with structured, mandatory second opinion for caesarean section indication is recommended to reduce caesarean births in settings with adequate resources and senior clinicians able to provide second opinion for caesarean section indication. *(Context-specific recommendation, high-certainty evidence)*

Recommendation 2.2. Implementation of evidence-based clinical practice guidelines, caesarean section audits and timely feedback to health-care professionals are recommended to reduce caesarean births. *(Recommended, high-certainty evidence)*

**Interventions targeted at health organizations or facilities**

Recommendation 3.1. For the sole purpose of reducing caesarean section rates, collaborative midwifery-obstetrician model of care (that is, a model of staffing based on care provided primarily by midwives, with 24-hour back-up from an obstetrician who provides in-house labour and delivery coverage without other competing clinical duties) is recommended only in the context of rigorous research. This model of care primarily addresses intrapartum caesarean sections. *(Context-specific recommendation, low-certainty evidence)*

Recommendation 3.2. For the sole purpose of reducing unnecessary caesarean sections, financial strategies (such as insurance reforms equalizing physician fees for vaginal births and caesarean sections) for health-care professionals or health-care organizations are recommended only in the context of rigorous research. *(Context-specific recommendation, very low-certainty evidence)*

WHO: World Health Organization.

## Implementation strategies

Countries should use a combination of interventions and strategies tailored to local determinants of inappropriate caesarean section use, for example the beliefs about the impact, professional norms and incentives, and economic and organizational factors influencing caesarean sections.

During implementation, monitoring and assessing caesarean section rates and maternal and perinatal outcomes is essential. This analysis should be done in a standardized and action-oriented manner, considering the specific characteristics of the populations served. For this purpose, WHO recommends the Robson Classification system as a global standard.^6^

Several factors may hinder uptake and scale-up of these new recommendations. These factors may be related to the behaviours or preferences of women and their families, the behaviour of health-care providers, the organization of care, health service delivery or financial arrangements. The barriers were identified from multiple sources: (i) qualitative reviews undertaken for this guideline;^7^^–^^9^ (ii) case studies and systematic reviews exploring factors affecting the implementation of interventions to reduce caesarean section rates;^10^^,^^11^ and (iii) Cochrane overviews of reviews of health-system implementation, care delivery arrangements and financial strategies.^12^^–^^14^ The barriers and proposed implementation strategies to overcome them are outlined below.

### Patient factors

These factors involve the lack of understanding of the value of recommended practices among women seeking maternity care, families or communities. To address this gap, a suggested strategy is to undertake community-level sensitization to disseminate information about the risks of unnecessary caesarean sections and the benefits of adhering to recommended practices. Such strategy should be delivered in a clear and consistent format using locally appropriate information, education and communication materials and activities.

### Health-care providers factors

Several factors can hinder implementation of the guideline’s recommendations. First, resistance of health-care providers to changing their practices coupled with a lack of understanding of the value of newly-recommended interventions. Suggested strategies to address this barrier include: involving local opinion leaders or champions to promote the implementation of the guideline’s recommendations; providing information or education that helps the targeted health-care providers to fit the recommended behaviour into their current practice; and involving training institutions and professional bodies in implementing the guideline so that pre-service and in-service training curricula can be updated with the recommendations. Successful implementation strategies should be documented and shared as examples to other implementers. Second, the working arrangements and numbers of senior clinicians (obstetrician-gynaecologists) may hinder the implementation of mandatory second opinion for caesarean section indication. This challenge can be addressed with a strategy that involves developing local case-specific protocols to ensure timely and appropriate senior clinician review for caesarean section indication and by organizing teams with defined roles and a shared goal towards reducing unnecessary caesarean sections. Third, patients may make demands for caesarean sections that hinder adherence to recommended evidence-based practices, which could be addressed by providing tailored patient education materials and training health-care professionals to provide patient education. Fourth, financial incentives may hinder adherence (for example higher pay for caesarean sections compared with vaginal births). One strategy to address this could be to remove or modify financial incentive by involving key stakeholder groups, including policy-makers and medical insurance agencies. Fifth, dysfunctional teamwork among health-care providers, such as lack of communication between maternity and operating room staff, and on occasion, difficult relationships between obstetricians, midwives and family doctors can hinder implementation of guideline recommendations. A suggested strategy would be to organize teams with defined roles and a shared goal towards reducing unnecessary caesarean sections.

### Health-care organization factors

Four factors belong to this category. First, lack of staff with the necessary expertise and skills to implement, supervise and support recommended practices. To address this shortage, a possible strategy would be to redistribute health-care resources, where and when possible, and to consider task shifting of roles where appropriate. Strategic long-term planning and budgeting to provide the necessary resources should also be contemplated. Second, lack of physical space, for example a venue for birth preparation classes, counselling sessions and training workshops. A proposed strategy to address this barrier is to adapt implementation strategies to work within the constraints of the existing systems and engage in strategic long-term planning and budgeting to provide the necessary resources. Third, lack of essential supplies including locally adapted information, education and communication materials to support training. Devising strategies to improve supply chain management according to local requirements, such as developing protocols for obtaining and maintaining the stock of supplies, is one of the suggested solutions. Fifth, lack of health information management systems designed to document and monitor recommended practices, which could be addressed by providing appropriate incentives to record the needed information. In this case as well, strategic long-term planning and budgeting to provide the necessary resources for health information management systems should be undertaken.

## Future research

The evidence underpinning the new recommendations was drawn from single studies with largely small samples. Therefore, the reported effect estimates and applicability of interventions should be confirmed in other settings, preferably in multicentre randomized trials. Future studies should be preceded by formative research to define locally relevant determinants of caesarean births that can be targeted by tailored interventions. The formative research would thus provide an important opportunity to modify and adapt the interventions to address key determinants of inappropriate caesarean use and achieve best fit with national policies and health system capacities, for example resources available.

Combinations rather than single-standalone interventions to reduce unnecessary caesarean sections are preferred, given the multiple determinants and stakeholders involved in decision-making about the mode of birth. Understanding of views, values and preferences, and active engagement of all stakeholders through participatory approaches is crucial throughout the process.

During pregnancy, women and the health-care providers need to interact, occasions which offer multiple opportunities to support informed decision-making around choice of the mode of birth. More research is needed to understand determinants of birth choices, so that the content and format of educational interventions can be tailored to relevant determinants of caesarean births. Prioritized research gaps are outlined in the full guideline.^4^

## Conclusions

Reducing unnecessary caesarean sections requires interventions that address both the clinical and non-clinical drivers of overuse. Clinical interventions that could help to reduce caesarean section rates have been addressed in previous WHO guidelines. The new guideline, targeting non-clinical drivers, should be implemented alongside existing WHO-recommended clinical interventions as part of an integrated approach to optimize the use of caesarean sections. Multifaceted strategies selected based on local determinants of caesarean section practices and health system capacities are recommended to address barriers and help improve successful implementation of the recommendations.
